# Efficacy and tolerability of mogamulizumab in mycosis fungoides and Sézary Syndrome: a monocentric retrospective study

**DOI:** 10.3389/fonc.2025.1748746

**Published:** 2026-01-22

**Authors:** Antonio Giordano, Luana Fianchi, Marianna Criscuolo, Martina Quattrone, Alessia Di Pilla, Livio Pagano

**Affiliations:** 1 Department of Hematology, Fondazione Policlinico Universitario Agostino Gemelli – IRCCS, Rome, Italy; 2 Università Cattolica del Sacro Cuore, Rome, Italy

**Keywords:** cutaneous lymphoma, mogamulizumab, monoclonal Ab, Sezary Syndrome, skin

## Abstract

**Background:**

Mycosis Fungoides (MF) is a common subtype of primary cutaneous T-cell lymphoma (CTCL), a group of non-Hodgkin lymphomas. The clinical spectrum of MF ranges from isolated cutaneous lesions to widespread involvement of lymph nodes, blood, and skin, as seen in its aggressive variant, Sézary Syndrome (SS). Mogamulizumab, a defucosylated humanized IgG1-κ anti-CCR4 monoclonal antibody approved for relapsed/refractory MF/SS, has demonstrated a favorable safety and efficacy profile in multiple case series.

**Methods:**

This retrospective, monocentric observational study analyzed data from 12 patients treated with Mogamulizumab between January 1, 2019, and December 31, 2024. We aim to evaluate the tolerability and clinical response to Mogamulizumab in patients with MF/SS.

**Results:**

Of the 12 patients treated, 8 had MF and 4 had SS. The median follow-up time was 29.9 months (range 2.8–68.6 months). Four patients discontinued mogamulizumab: 3 due to disease progression and 1 due to the development of breast cancer. Adverse events included MAR in 4 patients (33%) and colitis in 1 patient (6%). The observed median PFS after mogamulizumab therapy was 5.4 months, and the observed ORR was 50%. For all 12 patients, the median time to response (TTR) was 129 days. The observed median overall survival (OS) was 11.5 months, with 1 reported death due to septic shock in a patient who underwent salvage allo-HSCT after mogamulizumab failure.

**Conclusions:**

The results of this study reaffirm the efficacy of Mogamulizumab therapy for patients with Mycosis Fungoides and Sézary Syndrome in a real-world setting, which involves treatment decisions that must often consider patient heterogeneity, comorbidities, and prior lines of therapy.

## Introduction

Primary cutaneous lymphomas (PCLs) represent a heterogeneous group of non-Hodgkin lymphomas (NHLs) with skin involvement at diagnosis (1). In Western countries, the estimated annual incidence of PCLs is approximately 1 per 100,000 individuals (2). Cutaneous T-cell lymphomas (CTCLs) constitute approximately 75–80% of all PCLs, whereas cutaneous B-cell lymphomas account for the remaining 20–25%². The classification currently in use is the 2022 edition of the World Health Organization Classification of Haematolymphoid Tumours (3).

Mycosis fungoides (MF) and Sézary syndrome (SS) are the most common CTCL subtypes, with an annual incidence of 4.1 cases per 1,000,000 for MF and 0.1–0.3 cases per 1,000,000 for SS (4). MF and SS have long been considered two stages of the same disease, with SS regarded as the leukemic variant of MF (5). Although both are characterized by clonal proliferation of CD4^+^, CD45RO^+^, CLA^+^ T lymphocytes with cerebriform nuclei, recent comparative genomic hybridization and gene expression profiling studies have shown that the two diseases originate from distinct T-cell subsets—MF from effector T cells and SS from central memory T cells (6, 7).

MF, including its variants folliculotropic MF (FMF), pagetoid reticulosis (PR), and granulomatous slack skin (GSS), accounts for approximately 60% of all CTCL cases (8). It arises from the neoplastic transformation of skin-homing CD4^+^ T lymphocytes expressing high levels of cutaneous lymphocyte antigen (CLA) and the chemokine receptors CCR4 and CCR7, which are involved in T-cell homing. Clinically, MF is characterized by an epidermotropic infiltrate of CD4^+^ T cells, which may present as patches, plaques, or tumors, typically following an indolent course (9).

CCR4 is strongly expressed by both MF and SS cells and represents an important therapeutic target. Optimal treatment for MF and SS depends on disease stage and may involve either topical or systemic approaches. Hematopoietic stem cell transplantation (HSCT) currently remains the only potentially curative option for MF; however, its feasibility is limited by patient age and comorbidities. Moreover, patients with MF frequently develop resistance to available treatments, highlighting the need for novel therapeutic strategies.

In early-stage MF, skin-directed therapies (SDTs)—such as topical corticosteroids, PUVA/UVB phototherapy, or skin irradiation—are recommended. Advanced-stage MF often requires systemic therapies, including oral bexarotene or gemcitabine. Among systemic approaches, immunotherapy has demonstrated meaningful activity in advanced MF/SS. Alemtuzumab (anti-CD52) (10) and pembrolizumab (anti–PD-1) (11) have shown overall response rates (ORR) of approximately 40%.

The defucosylated humanized IgG1-κ monoclonal antibody mogamulizumab was first approved in Japan in 2012 for adult T-cell leukemia/lymphoma (ATLL) and peripheral T-cell lymphoma (PTCL), and later in 2018 in the United States and Europe for relapsed or refractory MF/SS (MF stage III–IV after one prior systemic therapy, or MF stage IB–II after at least two prior systemic treatments). Mogamulizumab selectively targets CCR4, mediating antibody-dependent cellular cytotoxicity (ADCC) and enhancing CD8^+^ cytotoxic T-lymphocyte (CTL) activity against skin-homing malignant T cells.

The MAVORIC trial, which compared mogamulizumab with vorinostat, a histone deacetylase inhibitor (HDAC) widely used in CTCL, demonstrated that mogamulizumab provided superior progression-free survival (PFS), ORR, and an improved safety profile (12).

Mogamulizumab is generally well tolerated, with infusion-related reactions and drug eruptions being the most common, usually mild, adverse events. However, mogamulizumab-associated rash (MAR) is the main adverse effect leading to treatment discontinuation (12). The pathogenesis of MAR appears to involve mogamulizumab-induced depletion of Th2 and regulatory T cells, which express CCR4 on their surface, resulting in immune dysregulation and pro-inflammatory activation of skin-homing CD8^+^ T cells (13). MAR may present with heterogeneous clinical patterns, including psoriasiform or morbilliform eruptions, photosensitive dermatitis, or alopecic scalp plaques. Distinguishing MAR from disease progression can be challenging; thus, histopathological confirmation is sometimes required to avoid inappropriate discontinuation of therapy. MAR typically occurs 2–6 months after treatment initiation. Interestingly, in some series, MAR has been associated with improved long-term clinical and hematologic responses (13).

Currently, real-world evidence on the effectiveness and tolerability of mogamulizumab in MF/SS remains limited. This monocentric real-life study aims to assess the efficacy and safety of mogamulizumab in 12 patients with MF/SS.

## Materials and methods

This retrospective, monocentric observational study analyzed data from 12 patients treated with Mogamulizumab between January 1, 2019, and December 31, 2024.

The patient staging was conducted based on the WHO-EORTC criteria (8), while treatment response was defined according to the standardized response criteria as described by the Tri-society consensus (14). The performance status of the patients was defined based on the ECOG (Eastern Cooperative Oncology Group) Score.

Mogamulizumab was administered intravenously at a dosage of 1 mg/kg over a minimum infusion time of 60 minutes. Each cycle lasted 28 days; for the first cycle, Mogamulizumab was infused on days 1, 8, 15, and 22 and from the second cycle only days 1 and 15.

Adverse events were categorized according to the Common Terminology Criteria for Adverse Events (CTCAE). The occurrence of MAR was defined based on clinical or histological features.

At the beginning of therapy with Mogamulizumab, the disease burden was evaluated using several parameters: these included metabolic tracer uptake measured through a PET-CT examination with FDG, immunophenotypic typing of peripheral blood, and TCR clonality analysis. For the assessment of blood involvement, the TNMB system criteria were applied, where a peripheral blood count of cells with a CD4^+^CD7⁻ or CD4^+^CD26⁻ phenotype exceeding 250/μL is considered positive. However, in certain pretreated patients with pronounced lymphopenia, the TCR molecular marker was employed to evaluate staging and to monitor minimal residual disease.

Patient characteristics are summarized in **Table 1**.

**Table 1:** Baseline patients’ and lymphoma characteristics.

Patients’ characteristics	Patients (n)	(%)
Patients (n)	12	
Sex (n)(%)		
M	7	(58,3)
F	5	(41,6)
Age (median)	74,5 (39-86)	
ECOG		
0	5	(41,6)
1	6	(50)
2	1	(8,3)
Lymphoma characteristics		
Mycosis fungoides (MF) (n)(%)	8	(66,6)
Sezary Syndrome (SS) (n)	4	(33,3)
LDH > 250 (n)	2	(16,6)
Blood involvement at diagnosis (n)	6	(50,0)
Stage (n)		
II B	3	(25)
IIIA	1	(8,3)
IIIB	3	(25)
IVA	3	(25)
IVB	2	(16,6)

### Statistical analysis

To assess the correlation between time to next treatment (TTNT) and the variables selected for statistical analysis, the nonparametric Spearman’s rank correlation coefficient (“rho”) was employed as a measure of monotonic dependence (i.e., an increasing or decreasing relationship that is not necessarily linear) between two variables, ranging from –1 to 1. The corresponding p-value was used to determine the statistical significance of the observed correlations.

**Figure 1:**
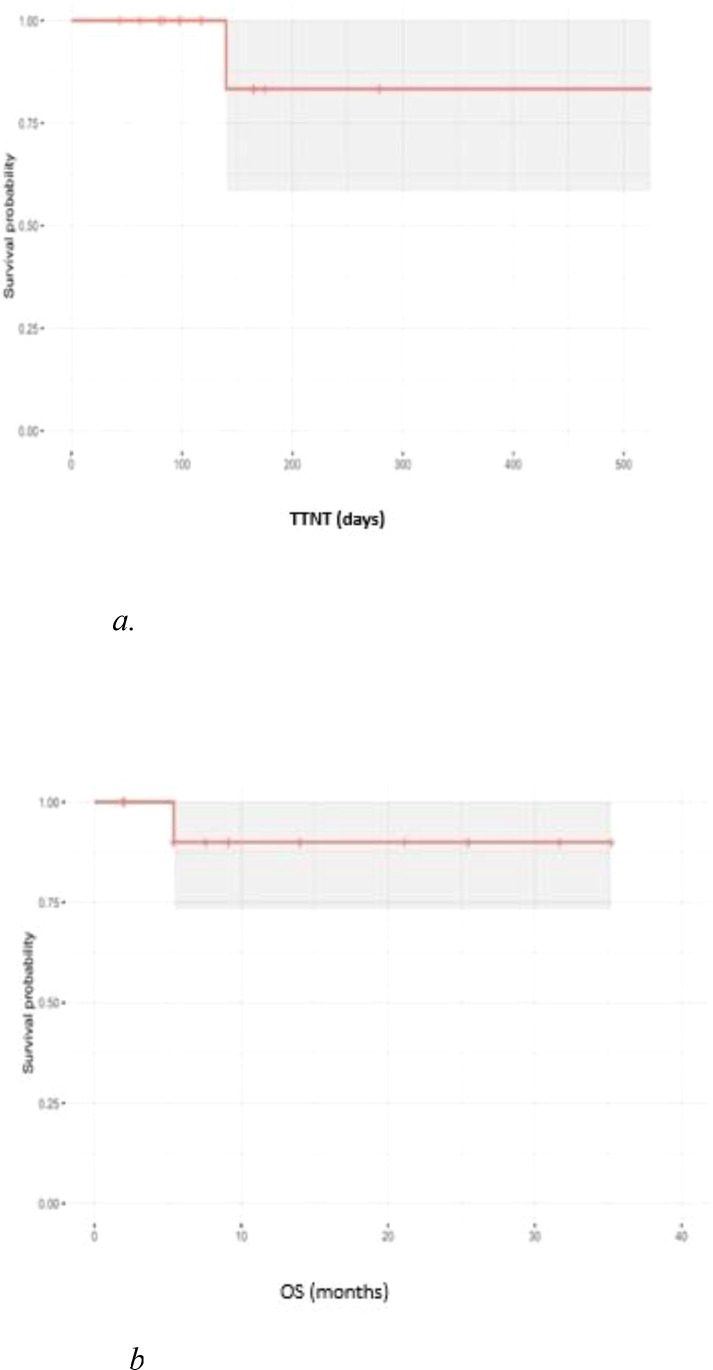
**(a)** Time to next treatment. **(b)** Overall survival.

For survival analysis, Kaplan–Meier curves were generated to estimate the median Time to Next Treatment (TTNT) and Overall Survival (OS) as shown in **Figures 1a, b**, respectively.

## Results

Among the 12 patients treated with mogamulizumab at our center, 8 had Mycosis Fungoides (MF) and 4 had Sézary Syndrome (SS). The median follow-up time was 29.9 months (range, 2.8–68.6 months).

Seven of the 12 patients underwent PET-CT at diagnosis, and 4 of them demonstrated positive lymph node uptake.

Peripheral blood involvement was assessed in all patients by flow cytometry and PCR analysis for T-cell receptor (TCR) clonality. A monoclonal TCR rearrangement was detected in 6 of 12 patients, and peripheral blood involvement was identified by flow cytometry in 6 patients. Three patients exhibited both a monoclonal TCR rearrangement and flow cytometric evidence of peripheral blood involvement. Two of these patients had the highest burden of circulating Sézary-like cells, fulfilling the B2 criterion—defined as ≥ 1,000/µL of aberrant CD4^+^CD7⁻, CD4^+^CD26⁻, or other phenotypically abnormal T-cell populations by flow cytometry in the presence of a confirmed T-cell clone; or alternatively, ≥ 1,000 Sézary cells/µL, CD4/CD8 ratio ≥ 10, CD4^+^CD7⁻ ≥ 40%, or CD4^+^CD26⁻ ≥ 30%.

The median number of prior lines of therapy before initiating mogamulizumab was 2 (range, 1–6). All patients had received at least one systemic treatment, with a median of 2 systemic regimens (including corticosteroids, bexarotene, and chemotherapy or conjugated antibodies such as gemcitabine, doxorubicin, or brentuximab vedotin) prior to anti-CCR4 therapy (range, 1–5) (**Table 2**).

**Table 2:** Treatment characteristics and response.

Prior lines of therapy	Number (n)	(%)
1	4	(33,3)
2	3	(25)
3	4	(33,3)
>3	1	(8,3)
Mogamulizumab therapy response		
CR	3	(25)
PR	3	(25)
SD	3	(25)
PD	3	(33,3)
MAR		
	4	(33,3)

Before starting mogamulizumab, 4 of 12 patients had received ≥ 3 lines of therapy, including at least 2 systemic regimens such as methotrexate, corticosteroids, and bexarotene. One patient had previously undergone 6 lines of therapy. Nine patients had been treated with oral bexarotene, 6 of whom discontinued due to intolerance (including dyslipidemia, hypertriglyceridemia, and hypercholesterolemia).

Four patients discontinued mogamulizumab: 3 due to disease progression and 1 due to the development of a solid neoplasm (breast cancer). One additional patient temporarily interrupted therapy after 4.2 months because of a suppurative skin lesion requiring antibiotic treatment but resumed therapy after 15 days.

In addition to infectious complications, other adverse events (AEs) included mogamulizumab-associated rash (MAR) in 4 patients (33%) and colitis in 1 patient (6%). The patient with colitis required hospitalization. Among those with MAR, the median onset was 189 days (range, 126–315 days), and the rash was graded as 1–2 in severity. Treatment consisted of topical corticosteroids in 1 patient and systemic corticosteroids in 3; none required discontinuation of mogamulizumab.

The median duration of mogamulizumab treatment was 5.23 months (range, 1.3–31.6 months), with a median of 5 cycles administered (range, 2–33).

The median progression-free survival (PFS) following mogamulizumab therapy was 5.4 months. The observed overall response rate (ORR) was 50%, comprising 3 complete responses (CR) and 3 partial responses (PR), while 6 patients had stable disease (SD) or progressive disease (PD).

Among the 3 patients who experienced disease progression after mogamulizumab, the median number of subsequent treatment lines was 3, including hematopoietic stem cell transplantation (HSCT) in 2 cases (16.6%).

For all 12 patients, the median time to response (TTR) was 129 days (**Figure 1a**). The median overall survival (OS) was 11.5 months, with one death due to septic shock in a patient who underwent allogeneic stem cell transplantation following mogamulizumab failure (**Figure 1b**).

## Discussion

Since the MAVORIC trial, several studies have highlighted the real-world efficacy of Mogamulizumab. Our real-life study further supports its favorable safety and efficacy profile, with 75% of patients demonstrating a clinical response—defined as complete response, partial response, or stable disease.

Similar to the cohort described in the study by Caruso et al. (15), our patients were refractory to first-line systemic therapy and presented with various stages of disease, starting from stage IIB.

Unlike their report, which described a case of SS with concurrent pancreatic adenocarcinoma, our patient with breast cancer had to discontinue treatment for the hematological disease in order to continue chemotherapy for the solid tumor. She remains under observational follow-up for MF.

The progression-free survival (PFS) in our cohort was 5.4 months, comparable to the PFS observed in the MAVORIC study (5.7 months). However, a retrospective multicenter study by the French group led by Jouandet et al. (16) involving 24 patients with MF/SS reported a significantly higher PFS of 22 months. More than 50% of the patients enrolled in the our study had a low performance status (ECOG 1–2), median age 74.5 and advanced-stage disease. Therefore, it is important to interpret overall survival within the context of a high-risk and more frail patients cohort. According to the staging criteria used (TNMB), the presence of blood involvement is indicative of an advanced stage. Therefore, it can be inferred that advanced-stage disease with a blood involvement and high tumor burden shows an excellent overall response to Mogamulizumab.

Despite the small sample size of our study and the lack of statistical significance, some variables appear to be associated with a favorable/unfavorable response trend. Specifically, the presence of a monoclonal TCR in peripheral blood correlates with a worsening of response time, as shown in **Table 3**.

**Table 3:** Statistical Analysis.

	rho	P-value	CI
Sex	0,269	0,397	-0,330; 0,780
TCR clonality	-0,531	0,075	-0,873; 0,000
ECOG	0,223	0,485	-0,466; 0,751
Stage	-0,021	0,947	-0,753; 0,622
LDH level	-0,259	0,416	-0,675; 0,255
Previous line Therapies	0,007	0,981	-0,607; 0,641
Sezary cells on peripheral blood	-0,416	0,178	-0,852; 0,253
OS	0,492	0,103	-0,252; 0,949

Consistent with other observational trials evaluating Mogamulizumab, the median time to response (TTR) in our study was 129 days (approximately 4.2 months), which is close to the 3.3 months reported in the MAVORIC trial and the 3.1 months in the OMEGA trial (17). This further confirms the rapid onset of the drug’s clinical effect.

Few studies have investigated the association between the onset of Mogamulizumab-associated rash (MAR) and clinical outcomes. In a retrospective case series of 24 patients conducted at the City of Hope Comprehensive Cancer Center, MAR developed in 68% of cases, with a mean onset of 2.1 months. Early MAR onset was correlated with improved overall response rates (ORR) (13). The high rate of MAR in that study may reflect close and meticulous patient monitoring, including photographic documentation and histological analysis of each new skin lesion.

The incidence of MAR in our cohort was also significantly lower (33%), with a median onset of 189 days—longer than the 107 days reported in the MAVORIC trial.

## Conclusions

Despite certain limitations related to the small sample size, the data presented herein reinforce the clinical utility of mogamulizumab as an effective and well-tolerated therapeutic option for patients with Mycosis Fungoides and Sézary Syndrome in a real-world setting. The agent demonstrated a manageable safety profile and achieved favorable disease control in a substantial proportion of treated individuals.

Moreover, a considerable subset of patients who initially experienced debilitating symptoms—including severe pruritus, pain, sleep disturbances, and fatigue—reported subjective symptomatic improvement over the course of treatment. However, due to the lack of validated quality-of-life assessment tools, these findings could not be objectively quantified or correlated with clinical endpoints.

Further data are needed to better elucidate the efficacy and safety profiles of mogamulizumab, particularly in the context of combination strategies (e.g., systemic chemotherapy in conjunction with skin-directed therapies), which are currently regarded as optimal approaches for disease management.

## Data Availability

The raw data supporting the conclusions of this article will be made available by the authors, without undue reservation.
